# “Mommy, do you love your phone more than me?”: Parental device use and the adolescent-caregiver attachment bond

**DOI:** 10.3389/fpsyg.2026.1766665

**Published:** 2026-06-18

**Authors:** Don Grant, Payne Winston-Lindeboom, Linda Ruan-Iu, Karen E. Shackleford, Barbara Nosal, Michael Roeske

**Affiliations:** 1 Newport Healthcare (Center for Research and Innovation), Nashville, TN, United States; 2 Institute for Graduate Clinical Psychology, Widener University, Chester, PA, United States; 3 School of Psychology, Fielding Graduate University-Santa Barbara, CA, United States

**Keywords:** adolescents, caregiver, device use, insecure attachment, media psychology, smartphones

## Abstract

While there is robust literature on the negative impact of adolescent device use on physical and psychological health, there is less research on the use of technology in the presence of others and its implications for key relationships. Known as “technoference” and “phubbing,” these device-based behaviors have only recently been examined in parent-child contexts. The present study investigated adolescents' perceptions of their primary caregivers' device-centric behaviors, the emotional appraisal of that behavior, and their association with the caregiver–adolescent attachment relationship. We hypothesized that adolescents' perceptions of less attentional availability would be associated with higher levels of insecure attachment. To test this, we validated the Device Attachment Interference Scale (DAIS) in a general population sample of U.S. adolescents (*N* = 600; ages 12–17). We also examined the association between DAIS scores and adolescent-reported attachment to a primary caregiver using the Experiences in Close Relationships–Relationship Structures scale. Exploratory and confirmatory factor analyses supported a unidimensional structure of the DAIS. Additionally, higher DAIS scores were consistently associated with greater insecure attachment (both anxious and avoidant) to both mother- and father-like figures. These findings highlight adolescents' perceptions of caregiver attentional availability in the context of device use as a potentially important relational context associated with attachment insecurity. Implications, limitations, and directions for future research are discussed.

## Introduction

Several years ago, a mother familiar with the first author's work surrounding healthy device use and digital-behavior mindfulness for families, shared with him a distressing event in which her young daughter asked her, “Mommy, do you love your phone more than me?” (Anonymous, personal communication, 2018). Similar accounts had been increasingly emerging in his clinical practice, with adolescents reporting that parental attention to screens during bids for connection left them feeling devalued, dismissed, or unimportant. Although prior research has documented how digital media use may alter communication patterns between people (Amelia and Balqis, 2023; Strauss et al., 2025), far less is known about the effect of device use in the proximity of others. This is especially relevant to caregivers and whether screen use may impact relational dynamics with adolescents. In particular, whether it shapes youths' perceptions of caregiver attentional availability and responsiveness—an essential component of attachment security (Bowlby, 1969)—and ultimately affects that bond.

Smartphone use is ubiquitous. While data on parents is limited, many adults have acknowledged that their smartphone use interferes with time spent with their children; in 2020, 68% of parents reported being at least “sometimes” distracted by their phones when with their child (Auxier, 2020). Teens echo this experience. In a 2024 Pew survey, 46% reported a parent “at least sometimes gets distracted by their phone” during conversations (Anderson, 2024). Together, these data highlight a modern concern: parental attention is competed for-and often captured by-smartphones and other digital devices. Given the pervasiveness of such distraction, even a modest impact on parent-child interaction could have meaningful consequences. The present investigation addresses this gap by examining how adolescents' perception of caregiver attentional availability related to their device use is associated with attachment-relevant experiences within the family system.

While there is robust literature investigating adolescent device use and its relationship with their physical and psychological health and wellbeing (Vinayak et al., 2024), there is a smaller group of studies on the association between technological distractions and relationship quality. The first research in this area focused on romantic and peer relationships (McDaniel and Coyne, 2016). In this context, the terms “technoference” (a portmanteau of technology and interference; McDaniel and Radesky, 2018) and “phubbing” (a portmanteau of “phone” and “snubbing;” Al-Saggaf, 2022) arose. Technoference predicts lower relationship satisfaction and quality and greater conflict (e.g., McDaniel and Drouin, 2019; McDaniel et al., 2021). Another term, “absent presence,” (Gergen, 2002) describes when an individual is physically “present” in a social setting, but mentally “absent” due to their captivation by a technological device such as a smartphone.

Only recently has a widening body of research begun to explore the possible consequences of technoference/phubbing in parent-child relationships (Coyne et al., 2025). McDaniel and Radesky (2018) found a positive relationship between technoference and child behavior problems. Similarly, Pancani et al. (2021) found parental phubbing was negatively related to children's wellbeing and relationship quality. In addition, Holmgren et al. (2024) measured mothers' time spent on their phones while with their children, finding that mothers who used social media on their phones showed greater distraction. Wu et al. (2022, p. 132) even describe parental phubbing as a “new form of social neglect during parent-child interactions,” resulting in adolescents who feel rejected by parents and alienated from peers. Similarly, Dixon et al. (2023) found that adolescents who perceive their parents as preoccupied with phones report greater feelings of emotional neglect or insecurity.

Perhaps most concerning, parental phubbing has been linked to insecure attachment in Chinese adolescents (Niu et al., 2020; Xie et al., 2019), American children (Zayia et al., 2021), and an adolescent clinical population (Grant et al., 2026). Attachment theory (Bowlby, 1969) posits that infants are biologically predisposed to seek proximity to caregivers for protection and emotional regulation. Later, through repeated interactions, children internalize working models of self and others that guide expectations in other relationships (Bretherton and Munholland, 2008). These models generally correspond to secure or insecure (anxious, avoidant, or disorganized) attachment patterns (Ainsworth et al., 1978; Main and Solomon, 1990). If the child is consistently, responsively, and sensitively attended to, they develop a secure attachment to their primary caregiver and learn that they are important and that their needs will be met. Greater attachment security has been linked to greater wellbeing, interpersonal competence, and life satisfaction (Mónaco et al., 2019).

Insecure attachment is associated with a variety of mental health conditions, such as depression (Spruit et al., 2020), anxiety (Williams et al., 2017), and post-traumatic stress disorder (Marshall and Frazier, 2019). Specifically, adolescent insecure attachment can lead to a range of outcomes, including difficulties with emotional regulation, self-esteem, and conflict resolution (Sutiyo, 2018). Insecure attachment is also linked with lifelong struggles with trust (of both self and others) and building healthy relationships, and thus with social isolation, behavioral problems, aggression, and risky behaviors, including substance abuse (Flykt et al., 2021). In addition, an insecure attachment in adolescents is tied to anxiety-driven clinginess or avoidance-driven emotional distance later in life (Delgado et al., 2022).

While attachment styles tend to be relatively stable through adolescence, they are influenced by experience (Jones et al., 2018; Waters et al., 2000). Risk factors include inconsistent or unavailable parenting, mental or physical health struggles, addictive behaviors, internal family system ruptures, neglect, and abuse (Parolin and Simonelli, 2016; Turner et al., 2019). Each has been shown to have a negative unfavorable relationship with child attachment security (Doyle and Cicchetti, 2017; U.S. National Library of Medicine, 2015). Because adolescence is a period in which parent-child attachment remains vital to healthy development and occupies a place of relational importance (Mónaco et al., 2019), we wondered if contemporary challenges, such as parental distraction by digital devices (i.e., technoference or phubbing), could be associated with attachment insecurity in an American, adolescent population. Given its specific and subjective nature, we needed a measure that would capture their perceptions of caregiver attentional availability and be distinct from broader relationship satisfaction.

Currently, there are various scales designed to generally measure technoference and phubbing within relationships. These include the Partner Phubbing Scale (Roberts and David, 2016), and its modified version for parents (Pancani et al., 2021), the Scale of Phubbing and the Generic Scale of Being Phubbed (Chotpitayasunondh and Douglas, 2018), and the Technology Device Interference Scale (McDaniel and Coyne, 2016). Stockdale et al. (2018) later adapted McDaniel and Coyne (2016) technoference scale specifically for adolescents, examining how device use affects both the child and the quality of the parent-child relationship. Additionally, Zayia et al. (2021) used a brief four-item survey with a small sample of elementary-aged children to investigate how children's attachment to their mothers relates to their perceptions of parental technoference. While many of these measures demonstrate good psychometrics, few were developed for adolescents or address adolescents' perception of their primary caregiver's attentional availability related to their device use. And none of the existing research target a parent's device-centric behaviors as an attachment risk factor in adolescents.

## Current study

The current study builds on a previous investigation where the authors created and validated a measure, examining whether an adolescent clinical population perceived that their primary caregiver's device distraction interfered with their attachment security (Grant et al., 2026). This measure includes both behavioral items (caregiver using their phone) and emotional items (how they feel about caregiver device use), all of which measure the perception of caregiver attentional availability. In that study, the authors' reported evidence for the unidimensional structure of the Device Attachment Interference Scale (Grant et al., 2026). As predicted, higher scores on the DAIS were significantly correlated with greater insecure attachment (both avoidant and anxious) to mother figures. The same relationship did not exist for father figures; however, the relatively small number of fathers reported as primary caregivers suggested more data was needed.

In the current study, three phases were conducted using a general population of U.S. adolescents. The first goal was to examine the psychometric properties of the DAIS. The second was to examine the reliability and validity of the DAIS. And the third was to investigate the relationship between device attachment interference (measured by the DAIS), and parental attachment (measured by the Experiences in Close Relationships- Relationship Structure scale [ECR-RS]; Fraley et al., 2011). Two research questions were explored:

1. Does the DAIS demonstrate evidence of reliability and validity in a general population of adolescents?
2. Are higher scores on the DAIS associated with increased attachment insecurity?

We predicted that higher scores on the DAIS would be linked to greater insecure attachment patterns in the adolescent sample. Further, we predicted that this relationship will hold regardless of the gender of the caregiver.

## Methods

### Participants

In August of 2025, adolescents [*n* = 600; aged 12 to 17 (*M* = 14.45, *SD* = 1.69)] were recruited using Qualtrics, a cloud-based software platform. Among the participants, 75.5% identified as White, 13.8% Black/African American, 3.8% Asian, 3.5% Multiracial, 2% Unsure, 1% Native Hawaiian/Pacific Islander, 0.3% American Indian/Alaskan Native, and 17.7% Hispanic or Latino. About 47.8% identified as Female, 47.2% Male, 2% genderqueer or gender non-conforming, 1.7% preferred not to respond, 0.9% as transgender, and 0.5% identified as “other” gender identity. Participants reported their primary caregiver as either a mother-like figure (*n* = 450; mother, stepmother, adoptive mother, grandmother), father-like figure (*n* = 125; father, stepfather, adoptive father, grandfather), or “other” parental figures (*n* = 25; aunt, uncle). Due to insufficient sample size, we removed the “other” category from further analysis.

### Procedure

After recruitment and screening, participants were sent an email invitation (or prompted via a survey platform) to proceed to the survey. Through the Qualtrics platform, consent was first acquired from a parent or legal guardian for the child's participation and then assent was collected from the adolescent. Additionally, based on the preferences given to Qualtrics, recruitment for the study included adolescents between the ages of 12 and 17 who represented the general population in the U.S. The study inclusion criteria additionally included that participants could speak, write, and understand English.

### Instrument development

The authors proposed a theory regarding the potentially unfavorable impact of a parent's technoference and phubbing on an adolescent's attachment bond security; and if it might be added to the list of primary caregiver attachment security risk factors. Recognizing both an increase in clinical presentations at the programs affiliated with the authors, and alignment with the existing studies on device-based relationship interference, a research team was coordinated to create a brief measure to assess adolescents' perception of caregiver attentional availability related to their device use.

A pool of 27 items was drafted by two of the authors, a psychometrician and a media psychologist, practitioner, and researcher. Their goal was to reduce the new measure to minimize respondent burden and increase feasibility in clinical settings, but remain comprehensive enough to obtain the desired information. The scale was first completed by 239 participants currently residing at two different residential treatment sites. To examine the initial internal structure, an exploratory factor analysis (EFA) was computed. The new scale was then peer reviewed by a team of psychologists and researchers with expertise in device-centric behaviors, attachment, and psychometrics. The 15 retained items were then administered at five treatment sites (two additional facilities participated). The goal was to collect data on a non-overlapping sample of 440 participants to further investigate the psychometric properties using confirmatory factor analysis (CFA); the 15 items were preserved for a clinical population. After reviewing the measure for the current study, three items were then removed due to poor factor loading leading to a final, 12-item measure.

***Assessment***. All participants completed a demographic questionnaire, an attachment measure (Experiences in Close Relationships-Relationship Structure; Fraley et al., 2011), and the DAIS (Grant et al., 2026). The survey took approximately 5 to 10 min. All data were de-identified by Qualtrics before the research team conducted analyses. The current study was approved by the organization's internal Research Review Panel and the Advarra Institutional Review Board.

## Measures

### Demographics

Four demographic questions were asked at the beginning of the assessment. These included the participant's age (in years), race, ethnicity, and gender identity.

### Device Attachment Interference Scale (DAIS)

The DAIS is a 15-item self-report measure that assesses the adolescent's perspective of their primary caregiver's device-centric behaviors. Participants rated the frequency (0 = *never*, 1 = *rarely*, 2 = *sometimes*, 3 = *often*, 4 = *almost always*) of each item, where higher scores indicate a greater frequency of device-centric behaviors. Evidence of the internal structure of the DAIS was supported in a clinical population (Grant et al., 2026). Items assess adolescents' perceptions that their caregiver's attentional availability “negatively affects our relationship,” that their caregiver “does not pay enough attention to me because of their device use,” “ignores me when they are on their device,” and “seems inattentive due to their device use.” See Table 1 for all DAIS items.

**Table 1:** Factor structure of the DAIS.

Item	F1
My primary caregiver's device use negatively affects our relationship.	0.704
My primary caregiver does not spend enough time with me because of their device use.	0.703
My primary caregiver's device use is a problem.	0.725
My primary caregiver ignores me when they are on a device.	0.691
When my primary caregiver is physically attending an event that is important to me, they seem inattentive due to their device use.	0.680
My primary caregiver spends too much time on their device(s).	0.713
When I want my primary caregiver's attention, but they won't put down their device, it makes me feel unimportant.	0.706
My primary caregiver is on their devices when they should be doing other things.	0.677
When attending any social event, my primary caregiver seems inattentive due to their device use.	0.706
I feel angry when my primary caregiver won't put down their device.	0.680
My primary caregiver is on their devices when they should be spending time with me.	0.764
My primary caregiver and I have conflicts about their device use.	0.629
My primary caregiver models good device use behavior.^*^	−0.165
My primary caregiver makes time for me even when they are on their devices.^*^	−0.218
My primary caregiver is typically available when I need them.^*^	−0.228

^*^indicates items that were eliminated from the final DAIS measure.

### Experiences in Close Relationship Scale-Relationship Structure (ECR-RS)

The ECR-RS (Fraley et al., 2011) was developed to measure attachment style and has been used with adolescent populations (Donbaek and Elklit, 2014). The 9-item self-report tool assesses avoidant and anxious attachment in close relationships. In the current study, participants selected a primary caregiver and then rated their relationship with them using a 7-point Likert-type scale (*strongly disagree* to *strongly agree*). Mean scores were generated for both attachment styles, where a higher score indicated greater endorsement of that insecure attachment style. The present study's cronbach's alphas were 0.87 (anxious) and 0.75 (avoidant) for father-like figures, and 0.89 (anxious) and 0.83 (avoidant) for the mother-like figure.

## Data analytic plan

EFA (*n* = 200) and CFA (*n* = 400) were conducted using two non-overlapping samples to examine the factor structure of the DAIS. For EFA, principal axis factor extraction was employed (and promax rotation was used when exploring multiple factors). To determine the number of factors, multiple methods were utilized including visual inspection of scree plot, parallel analysis, and MAP analysis. A priori criteria for factor retention include a factor loading of ≥0.40, at least three items per factor, and the factor is theoretically meaningful. Internal consistency was estimated using Cronbach's alpha for non-bifactor models. CFA was then computed using maximum-likelihood in Mplus version 8 (Muthén and Muthén, 2017). Model fit indices used to determine model fit includes Root Means Square Error of Approximation (RMSEA; Steiger and Lind, 1980) ≤ 0.08, Comparative Fit Index (CFI; Bentler, 1990) ≥0.95 (Hu and Bentler, 1999), and a non-significant Chi-Square Goodness of Fit. Finally, regression analyses were used to examine whether device-centric behaviors (DAIS) were associated with attachment (ECR-RS) to mother-like figures and father-like figures, controlling for age and gender.

## Results

### Internal structure

**Figure 1:**
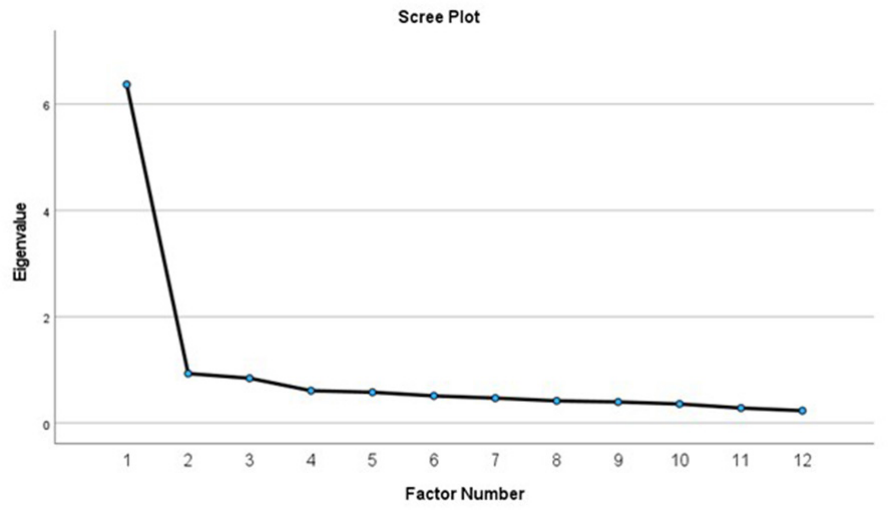
Scree plot for DAIS factor structure.

Kaiser-Meyer-Olkin Measures of Sampling Adequacy and Bartlett's Test of Sphericity [KMO =0.923; χ2 (66) = 1231.416, *p* < 0.001] suggested the items are sufficiently correlated and are suitable for factor analysis. EFA results (scree plot, parallel analysis, and MAP) supported a unidimensional factor structure (see Figure 1). A total of 15 items were entered, and 3 items did not meet a priori criteria of having at least a 0.40 loading, and thus, removed. A final model consisted of 12 items, and the single factor (α = 0.919) appears to assess device centric behaviors (see Table 1). The one-factor model accounted for 53% of the variance. The initial eigenvalues indicate that only one factor had an eigenvalue >1 (λ = 6.370), with all subsequent factors eigenvalues below 1 (0.931 to 0.232). Communalities range between 0.396 to 0.583. Thus, the one-factor model was retained and then examined using CFA. Results suggest an acceptable model fit [χ2(66) = 1658.742, *p* < 0.001, CFI = 0.931, RMSEA = 0.072 (90% CI = 0.059,0.084)]. While the one-factor model did not exceed a priori criteria CFI> 0.95 (Hu and Bentler, 1999), it yielded an acceptable model fit (CFI >0.90; Little, 2013; van Laar and Bracken, 2021), and thus, was retained for further analysis. No modifications of the model were made.

### Regression analysis

To investigate whether the primary caregiver's device-centric behavior was associated with attachment, regression analysis was computed, controlling for demographics. Regression analyses were computed separately for mother-like figures and father-like figures due to unequal sample sizes between the two groups. Multicollinerity was examined using VIF, and they were within acceptable limits (VIF < 2) for all models. Regression results indicated that higher device-centric behaviors were related to more avoidant (*b* = 0.630, *SE* = 0.077, *t* = 8.180, *p* < 0.001) and anxious (*b* = 0.819, *SE* = 0.087, *t* = 9.360, *p* < 0.001) attachment with mother-like figures. Results were similar with father-like figures and showed that elevated caregiver device use was related to more avoidant (*b* = 0.434, *SE* = 0.101, *t* = 4.311, *p* < 0.001) and anxious (*b* = 0.990, *SE* = 0.105, *t* = 9.399, *p* < 0.001) attachment.

## Discussion

The purpose of this study was to investigate adolescents' perceptions of their primary caregivers' attentional availability related to their device use and the association to attachment security in a U.S. representative sample. Through examination of the internal structure of the DAIS, findings supported a unidimensional structure. Additionally, we observed a pattern of association between higher DAIS scores and more insecure (both anxious and avoidant) attachment to both mother- and father-like figures. Interestingly, in the original study (Grant et al., 2026), increased caregiver device-centric behaviors were only significantly associated with more insecure attachment (both anxious and avoidant) with mother-figures. Thus, the current investigation adds to the previous study which may have suffered from an insufficient sample size to detect this issue in fathers. Or it may have revealed a difference between general and clinical populations. Overall, this data is evidence that higher caregiver device-centric behaviors have a relationship with problematic family dynamics, specifically those related to insecure attachment.

This study does not come without limitations. First, it is important to recognize that the present findings are correlational and cross-sectional in nature and therefore do not permit conclusions regarding directionality or causality. Although caregiver device-centric behaviors and adolescent attachment insecurity were robustly associated, it remains unclear whether perceived caregiver distraction is related to attachment insecurity, whether adolescents with higher levels of attachment insecurity are more likely to perceive caregiver behavior as inattentive or disruptive (i.e., reverse causality), or whether both reflect other unmeasured contextual or relational factors. Second, several of the DAIS items assess emotional reactions, which could conceptually overlap with anxious attachment. Thus, the relationship between the DAIS and ECR may reflect a partial overlap of the constructs. Third, both the DAIS and ECR-RS are self-reported measures at a single time point, which may have shared method variance. Fourth, we did not have a large enough sample size to perform measurement invariance testing, which will allow for comparison of mother and father-like caregiver scores, and would be valuable in future studies. Additionally, while multicollinearity was assessed, additional assumption testing was not included. Fifth, as we have noted throughout, the DAIS items measured both 1) adolescent's perception of caregiver's device-related behaviors and 2) the adolescents' emotional appraisal of that behavior. Our analyses revealed that adolescents' perceptions of caregivers' device use and the way that device use affects the adolescent-caregiver relationship can be captured as one latent construct. However, because this is the first published research on the scale's use, future research may confirm a single-factor solution, or a two-factor solution may emerge in some instances. Sixth, although we have evaluated the newly-created DAIS through its internal structure and its association with insecure attachment, we suggest remaining cautious about its validity until future research establishes convergent and discriminant validation with other related constructs.

Finally, adolescents with insecure attachment orientations may also be more sensitive to perceived parental unavailability, including device-related distraction, and thus may interpret caregiver behavior more negatively. Longitudinal research, as well as multi-informant and multi-method approaches would assist in disentangling these possibilities. Because device-centric behaviors such as parental distraction and insufficient responsiveness to adolescents' bids for attention likely occur multiple times a day and take place in the natural environment, clarifying directionality and underlying mechanisms creates additional challenges. One possible starting point for providing useful data is video recording of parent-adolescent interactions with devices present. These interactive sessions could be followed by reviewing the interaction with the adolescent and establishing what the adolescent considers low and high-quality parental responses. Additionally, viewing the above-described parent-adolescent interactions could increase awareness in both parents and adolescents of potentially problematic behaviors. This technique could be used in general populations, such as the one studied here.

Such research could extend to clinical populations and thus be potentially useful in therapeutic contexts, as the DAIS may also have practical utility in assessment settings. In particular, it may aid in identifying patterns of perceived caregiver inattention that may be relevant to family relationships and particularly attachment-related concerns. These findings may support conversations with families about device use in relational contexts, an area that is often under-addressed in both public discourse and clinical practice. Until future research provides more data about the validity of this new instrument, any conclusions about the scale's clinical applications should be made cautiously.

## Conclusion

Given the high prevalence of device use among adults, even modest associations at the individual level may have broader implications at the greater population level. These findings align with a growing literature on technoference and phubbing, which has documented associations between device-related distraction and relationship quality across a range of interpersonal contexts. These findings may also contribute to attachment theory by highlighting adolescents' perceptions of caregiver attentional availability—specifically in the context of device use—as a potentially important context associated with attachment insecurity. Unlike traditional forms of caregiver unavailability, device-related distraction is often intermittent, socially normalized, and embedded within otherwise typical caregiver–adolescent interactions. Also, unlike other caregiver attachment risk factors identified above (e.g., inconsistent or inaccessible parenting, caregiver mental or physical health difficulties, addictive behaviors, disruptions within the family system, neglect, or abuse), device-related behaviors are entirely subject to the caregiver's volitional control. As such, even brief but repeated disruptions in caregiver responsiveness may take on relational significance for adolescents. Taken together, the present findings highlight the importance of considering caregiver device use not only as an individual behavior, but as a relational context that may play an important role in adolescents' experiences of connection and attachment bond security.

## Data Availability

The raw data supporting the conclusions of this article will be made available by the authors, without undue reservation.
